# Hsu-Nielsen source acoustic emission data on a concrete block

**DOI:** 10.1016/j.dib.2019.103813

**Published:** 2019-03-06

**Authors:** Ramin Madarshahian, Vafa Soltangharaei, Rafal Anay, Juan M. Caicedo, Paul Ziehl

**Affiliations:** a Department of Structural Engineering, University of California at San Diego, USA; b Department of Civil and Environmental Engineering, University of South Carolina, Columbia, SC, USA

**Keywords:** Acoustic emission, Pencil lead break, Concrete block, Onset time

## Abstract

Data presented in this paper are utilized in the paper entitled “Acoustic Emission Bayesian Source Location: Onset Time Challenge” [1]. Hsu-Nielsen source which also known as pencil lead break (PLB), is an artificial method of generating acoustic emission (AE) signals, which can roughly represent an acoustic emission damage source. The data in this paper represent AE signals emitted by conducting PLBs on a concrete block. The test was repeated ten times for three different locations. The resulted stress waves were captured by piezoelectric acoustic emission sensors and acquired as the electrical signals. The signals were digitized according to a specified sampling rate and were presented as voltage amplitudes. Each PLB was registered by several sensors (data acquisition channels). The data are presented for each PLB and channel. Furthermore, the geometry and mixture design of the concrete block, sensor types, sensor locations, and PLB locations are reported. The data can be used for validation of source location algorithms, signal processing, and sensor calibration.

**Table:** Specifications table

Subject area	*Civil Engineering*
More specific subject area	*Structure health monitoring, nondestructive test, structural damage detection, structural condition assessment*
Type of data	*Table (Excel files)*
How data was acquired	*Piezoelectric acoustic emission sensors and acoustic emission data acquisition.*
Data format	*Raw Data*
Experimental factors	*Sensor attachment, sensor calibration, and data acquisition setup*
Experimental features	*The AE sensors were attached on the specimen using epoxy. The sensors were connected to the data acquisition using cables and the data acquisition setting and sensor calibration were conducted before collecting the data.*
Data source location	*Columbia, South Carolina, 29208,USA*
Data accessibility	*The data available with this article*
Related research article	R. Madarshahian, P. Ziehl, J.M. Caicedo, Acoustic emission Bayesian source location: Onset time challenge, Mechanical Systems and Signal Processing, 123 (2019) 483–495 [1].

**Table:** 

**Value of the data** • The data illustrates artificial acoustic emission phenomena, Hsu-Nielsen source, in a concrete structure. This data can be used as a benchmark to validate the different source location algorithms in acoustic emission technology and even in seismology. The sensor arrangement enables the source localization verification in 1D, 2D, and 3D spaces. In addition to source location solver algorithm, the data can be used for verifying the algorithms to detect the time of arrival for elastic waves. • The data can be used to analyze the effect of wave propagation direction in source location or signal processing. • The raw data and frequency-domain version of the data can be used for developing wave propagation models. • The AE data can be utilized for researchers who are interested in sensor optimization.

## 1 Data

**Fig. 1:**
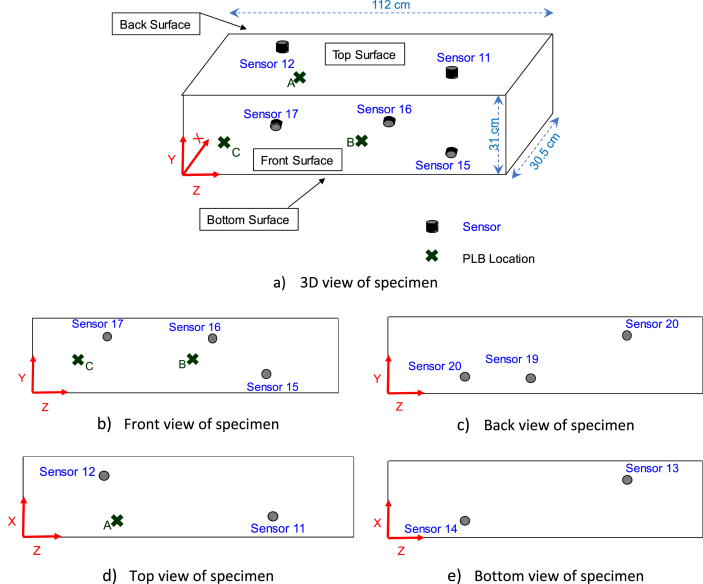
Specimen sensor layout and pencil lead break locations.

Source location or damage localization is one of the important goals in structural health monitoring and damage detection methods. Acoustic emission has a good potential to satisfy this goal. Elastic waves emitted from structural damage are detected by different AE sensors. The location of source can be identified based on the difference in arrival times at the individual sensors. The data presented is achieved by breaking 0.3 mm HB pencil leads on the surface of a concrete beam [2]. The pencil break resembles the real crack formation and acoustic emission phenomenon. The beam is shown in Fig. 1. The pencil breaks caused stress waves that propagated through the specimen and reached the piezoelectric sensors. The sensors changed the mechanical waves to electrical signals. The signals were amplified and digitized by the data acquisition system. Ten sensors were attached on the surface of the specimen as shown in Fig. 1. The coordinates of the sensors are presented in Table 1. Moreover, the locations of pencil lead breaks are indicated in the figure and provided in Table 2. The data are CSV files. Each file has a signal caused by a pencil lead break and captured by a sensor. Sensor names were labeled from 11 to 20 according to the associated channel number in the data acquisition system. The name format of the CSV files is PLBX_Y_Z. “PLB” stands for pencil lead break. “X” can be either A, B, or C (pencil lead break location). “Y” is the channel number (sensor number) related to a sensor and can be 11 to 20. “Z” is an arrival time for an event multiplied by 10ˆ6, captured by a sensor and calculated according to the device threshold. For instance, the file “PLB A_11_43561886” is related to pencil lead broken at location “A”, captured by sensor 11 and with the arrival time of 43.561886 seconds. Some information is included in each file such as test date, test time, sample interval, signal unit, number of data, and channel numbers. The sample interval is the time difference between two sequential data points in second. Signal unit is shown in Volt. Number of data is the number of captured points in each signal. The data start from line 13 of the files. This data can be useful for a source location algorithm or any other analysis methods for AE signal processing.

**Table 1:** Sensor location.

Sensor Number	X (cm)	Y (cm)	Z (cm)
11	8.5	31	84
12	24.3	31	27.9
13	23	0.0	83.8
14	7.6	0.0	27.0
15	0.0	6.5	84
16	0.0	23	64
17	0.0	23	27.5
18	30.5	23	85
19	30.5	7.0	48.5
20	30.5	6	28

**Table 2:** Pencil lead break locations.

PLB location	X (cm)	Y (cm)	Z (cm)
A	7.6	31.0	38.1
B	0.0	15.2	55.9
C	0.0	15.2	15.2

## 2 Experimental design, materials, and methods

A concrete specimen having dimensions of 30.5 cm wide, 31.0 cm high, and 112 cm long is used as the test specimen. The concrete mixture had a water to cement weight ratio of 50%. The concrete mixture included 350.0 kg/m^2^ Portland cement type II (according to ASTM C150), 1050 kg/m^2^ coarse aggregate (crushed Greenschist from Vulcan Gold Hill, North Carolina), 850.8 kg/m^2^ natural sand with quartz and Chert (from Robstown, Texas), and 1.026 kg/m^2^ NaOH aqueous solution (50% solution). The concrete block did not have any visual cracks or defects. Ten piezoelectric acoustic emission sensors were attached to the specimen. The sensors are PKWDI sensors with an operating frequency range of 200–850 kHz from MISTRAS (physical acoustics corporation). The sensors have low-power internal pre-amplifiers with 26 dB gain. The specimen and sensors are shown in Fig. 1. The coordinates of the sensors are illustrated in Table 2. The coordinates are based on the origin coordinate, indicated in the figure. The sensors are labeled according to the connected channel in the data acquisition and shown inside the ovals in the figure. Sensors 13 and 14 were located at the bottom of specimen, and sensors 18, 19, and 20 were located on the other side of the specimen.

Before attaching the sensors, the locations of sensors on the specimen surface were cleaned and smoothed. The epoxy was utilized as the coupling agent to fix the sensors on the surface of the specimens. The sensors were firmly held in place for epoxy curing. The holders were made from stainless steel to hold the sensors in their locations with pressure. The holders were also attached by epoxy. The sensors were connected to a 24-channel Express-8 AE data acquisition system manufactured by MISTRAS Group. The threshold of 31 dB was set to attenuate the background noise. The sampling rate was 1000 kHz. Peak Definition Time (PDT), Hit Definition Time (HDT), Hit Lock Time (HLT), and Pre-Trigger Time were 200 μs, 400 μs, 200 μs, and 256 μs, respectively. These are the waveform settings in the data acquisition system. Pre-trigger time is the time required for data acquisition to save a signal prior to threshold intersection. This time is important to find a correct time of arrival for a signal. PDT is the time constant of re-triggerable one-shot in the circuity that determines Rise time. HLT is a lockout time, starting at the end of hit, during which the system does not response to threshold crossings. HDT is the time constant of the re-triggerable one-shot circuitry that determines the end of hit [3], [4].

After the sensors and data acquisition setup, pencil leads (Hsu-Nielsen source [5]) with 0.3 mm diameter are broken ten times in each specific location (A, B, and C in Fig. 1). The waves that are emitted are captured by the sensors and recorded as digital signals. The locations of pencil lead breaks are shown in Fig. 1 and their coordinates are presented in Table 2.
